# Supplementary material for: Association Between Sarcopenia and Acupressure Testing in Older Adults Requiring Long-Term Care
Source: Medicina (Kaunas). 2024 Nov 11;60(11):1852. doi: 10.3390/medicina60111852 (PMC11596401; doi:10.3390/medicina60111852)
Supplement: Supplementary file 1 [file medicina-60-01852-s001.zip › medicina-3261580-supplementary.pdf]

medicina

MDPI

Supplementary Materials

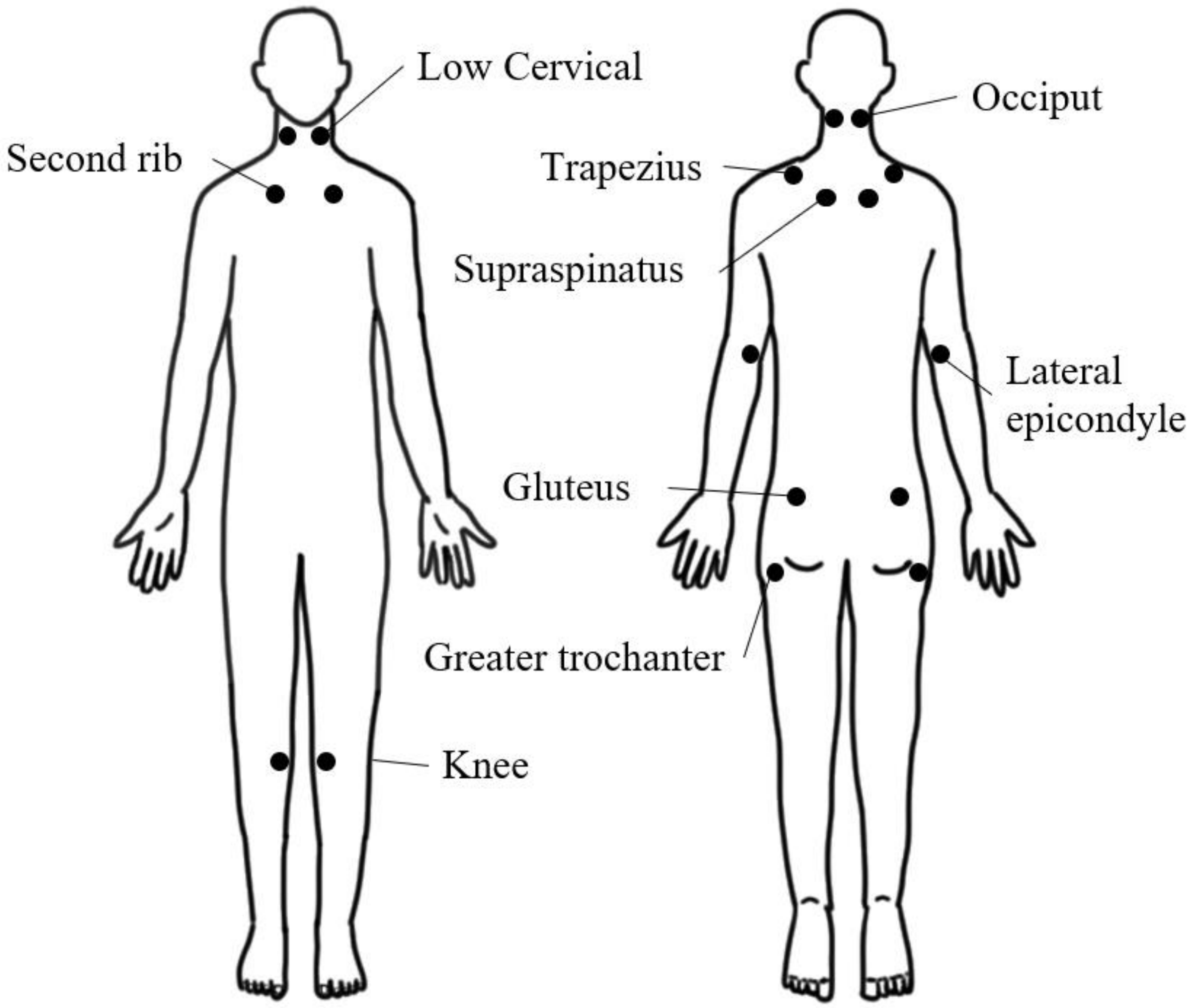

Figure S1. Sites of acupressure conducted in this study.

Table S1. Correlations between the number of total tender points and sarcopenia diagnostic items in males (n = 75).

	Total Number of Tenderness	SARC-F †	Grip Strength	Gait Speed	SMI	BMI	MNA-sf	Care Levels
Total number of tenderness								
SARC-F †	0.143							
Grip strength	−0.408 **	−0.048						
Gait speed	−0.214	−0.476 **	0.128					
SMI	−0.307 **	−0.045	0.554 **	0.144				
BMI	−0.196	−0.250 *	0.230 *	0.126	0.413 **			
MNA-sf	−0.373 **	−0.319 **	0.413 **	0.196	0.398 **	0.448 **		
Care levels	0.168	0.212	−0.131	−0.179	−0.022	−0.167	−0.225	

*: $p < 0.05$, **: $p < 0.01$. †: With missing date (n = 73). SMI: skeletal muscle mass index. BMI: body mass index. MNA-sf: Mini Nutritional Assessment Short-Form.

Table S2. Correlations between the number of total tender points and sarcopenia diagnostic items in females (n = 45).

	**Total Number of Tenderness**	**SARC-F**	**Grip Strength**	**Gait Speed**	**SMI**	**BMI**	**MNA-sf**	**Care Levels**
Total number of tenderness								
SARC-F	0.168							
Grip strength	−0.486 **	−0.175						
Gait speed	−0.280	−0.463 **	0.344 *					
SMI	−0.335 **	−0.104	0.580 **	0.437 **				
BMI	−0.109	0.097	0.075	0.128	0.523 **			
MNA-sf	−0.156	−0.170	0.138	0.060	0.492 **	0.327 *		
Care levels	0.255	0.088	−0.200	−0.565 **	−0.606 **	−0.228	−0.216	

*: $p < 0.05$, **: $p < 0.01$. SMI: skeletal muscle mass index. BMI: body mass index. MNA-sf: Mini Nutritional Assessment Short-Form.
